# Pleomorphic xanthoastrocytoma with anaplasia and BEND5-NTRK2 fusion in a young adult with a history of cranial radiation for childhood rhabdomyosarcoma

**DOI:** 10.1093/noajnl/vdaf052

**Published:** 2025-03-08

**Authors:** Julie M Fischer, Andrea R Gilbert, Eva M Galvan, Achint K Singh, John R Floyd, Shafqat Shah

**Affiliations:** Department of Pediatric Hematology and Oncology, University of Texas Health San Antonio & University Hospital, San Antonio, Joe R. & Teresa Lozano Long School of Medicine, San Antonio, Texas, USA; Department of Pathology and Laboratory Medicine, University of Texas Health San Antonio & University Hospital, San Antonio, Joe R. & Teresa Lozano Long School of Medicine, San Antonio, Texas, USA; Department of Radiation Oncology, University of Texas Health San Antonio, Mays Cancer Center, San Antonio, Texas, USA; Department of Neuroradiology, University of Texas Health San Antonio & University Hospital, San Antonio, Joe R. & Teresa Lozano Long School of Medicine, San Antonio, Texas, USA; Department of Neurosurgery, University of Texas Health San Antonio & University Hospital, San Antonio, Joe R. & Teresa Lozano Long School of Medicine, San Antonio, Texas, USA; Department of Pediatric Hematology and Oncology, University of Texas Health San Antonio & University Hospital, San Antonio, Joe R. & Teresa Lozano Long School of Medicine, San Antonio, Texas, USA

**Keywords:** anaplastic pleomorphic xanthoastrocytoma, next-generation sequencing, NTRK fusion, NTRK inhibitors, radiation-induced gliomas, secondary high-grade gliomas

**Pleomorphic xanthoastrocytoma (PXA) is a rare astrocytic tumor, with an anaplastic variant (PXA3) exhibiting more aggressive behavior and worse prognosis. We report the case of a young adult who developed PXA3 with a novel *BEND5-NTRK2* fusion 2 decades after receiving cranial radiation for childhood rhabdomyosarcoma. The tumor’s histopathology was challenging to classify, necessitating advanced molecular profiling, which confirmed the diagnosis. The identification of an *NTRK2* fusion enabled targeted therapy with Larotrectinib, leading to sustained tumor control without further radiation. This case highlights the significance of comprehensive molecular testing in rare pediatric and young adult gliomas.**

Pleomorphic xanthoastrocytoma, a grade 2 (PXA2) neoplasm by the World Health Organization (WHO) classification, is a rare subset of circumscribed astrocytic glioma.^1^ It represents <1% of astrocytomas and is commonly diagnosed in the 2nd decade of life.^2^ WHO grade 3 PXA (PXA3), historically referred to as anaplastic PXA, is a malignant PXA subtype typically defined by the presence of anaplasia, necrosis, high mitotic activity, and increased cellularity.^2^ While PXA2 typically has a favorable prognosis with a 5-year survival rate of >75%, PXA3 has a poorer prognosis with a 5-year survival rate of 57%.^2^ Molecular analysis of PXA2 and PXA3 often reveals *BRAF* mutations and/or *CDKN2A/B* homozygous deletions.^1–5^ Most analyses have been performed in adult cases and less is known about the molecular analysis of PXA2 and PXA3 tumors in pediatric and young adult patients. *NTRK* fusions have been identified in several types of pediatric gliomas, with an incidence of around 5% in pediatric high-grade gliomas and glioblastomas.^6^*BEND-5* gene alterations have rarely been reported in adult central nervous system (CNS) tumors such as glioblastoma but have not previously been reported in pediatric PXA2 or PXA3.^7^ We report a patient with a history of cranial radiation for rhabdomyosarcoma (RMS) who developed a PXA3 with *BEND5-NTRK2* fusion 20 years later.

## Case Presentation

**Figure 1.**
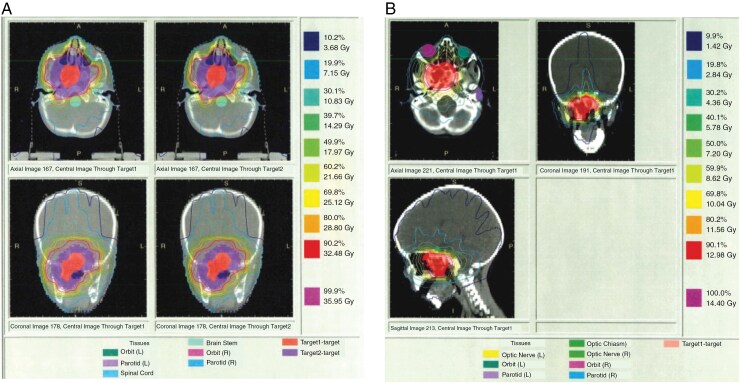
(A) Field of radiation of nasopharyngeal RMS to 36 Gray at age 3. (B) This was followed by a cone-down boost to 14.4 Gray (prescription) with the outermost line representing 1.4 Gray reaching the occipital lobe.

The patient initially presented to primary care at age 3 with right eye esotropia. She had recently been treated with amoxicillin for acute otitis media and had been experiencing 2 weeks of congestion, rhinorrhea, and eye pain. She had not experienced any neurologic symptoms to include headaches, weakness, dizziness, or changes in ambulation. In the emergency department, she had abnormalities on computed tomography (CT) and magnetic resonance imaging (MRI) imaging which identified a nasopharyngeal mass. This ultimately prompted a biopsy with pathology revealing nasopharyngeal (parameningeal) RMS, embryonal subtype. Molecular testing was not performed on the tumor. She was tested for Li-Fraumeni Syndrome and was reportedly negative. She was treated with vincristine, actinomycin, and cyclophosphamide per POG 9803. She also received cranial intensity-modulated radiation therapy at 50.4 Gray to the nasopharynx (Figure 1A and B). She had a favorable response to treatment and was followed until age 8 with serial imaging without evidence of disease recurrence. During this time, she was intermittently followed by endocrinology for short stature and gynecology for amenorrhea but otherwise remained in good health.

**Figure 2.**
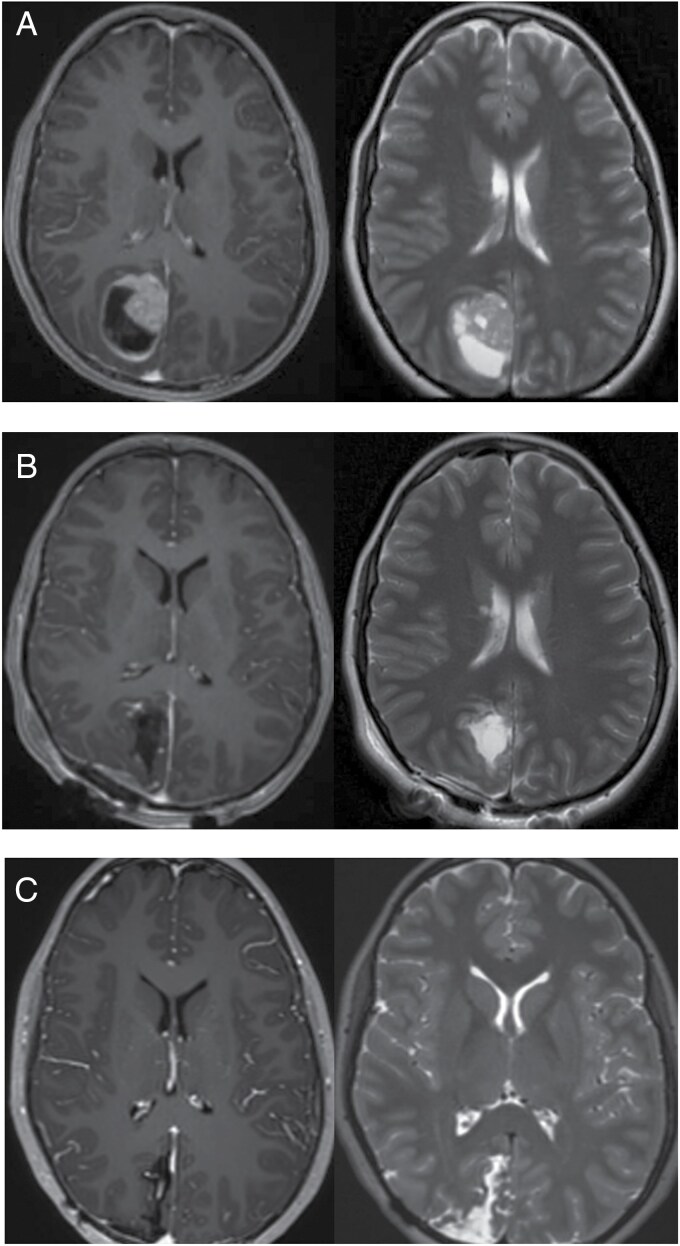
(A) Initial T1 enhanced (left) and T2 (right) MRI brain imaging revealing circumscribed right occipital lobe mass. (B) Post-resection T1 enhanced (left) and T2 (right) MRI brain imaging. (C) Most recent surveillance T1 enhanced (left) and T2 (right) MRI imaging.

At age 17, she had a CT head done following a motor vehicle accident and was told it was “abnormal” but “did not look like a tumor.” At age 22, she developed left eye visual changes and was evaluated by ophthalmology who diagnosed her with an ocular migraine. At age 23, she developed a severe headache and was evaluated in the emergency department where a CT head and MRI brain were done revealing a circumscribed mass in the right occipital lobe (Figure 2A). She underwent gross total resection (Figure 2B) and did well postoperatively.

**Figures 3.**
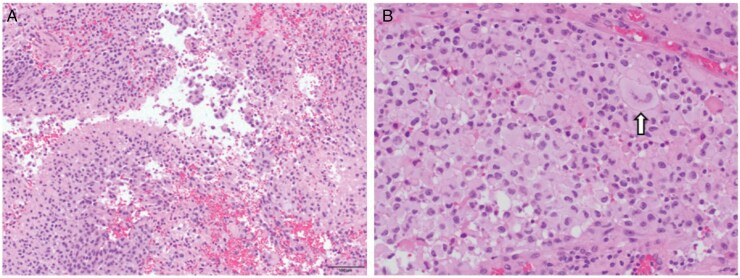
(A and B) Hematoxylin and eosin-stained sections show a high-grade glioma with neoplastic cells exhibiting prominent nuclear atypia and pleomorphism (arrow) and epithelioid cytomorphologic features characterized by large central nuclei, ample cytoplasm, and distinct cell borders. (magnification: A-100×, B-200×)

Microscopic evaluation of the resected tissue revealed a high-grade glioma with focal necrosis, nuclear pleomorphism, and epithelioid cytology featuring cells with large, rounded nuclei, ample semi-glassy cytoplasm, and distinct cell borders (Figure 3). Immunohistochemistry (not shown) for MIB-1 revealed an elevated proliferation index and PHH3 revealed frequent mitoses. Tumor cells were loosely arranged in lobules with intervening septa containing hyalinized vessels with focal vascular proliferation. Morphologically the tumor was difficult to classify, and diagnoses considered in the differential included epithelioid glioblastoma multiforme (eGBM), WHO grade 4, as well as the epithelioid variant of PXA3, though, notably, many classic histopathologic PXA features, including eosinophilic granular bodies and xanthomatous cells, were not detected in this specimen.

**Figure 4.**
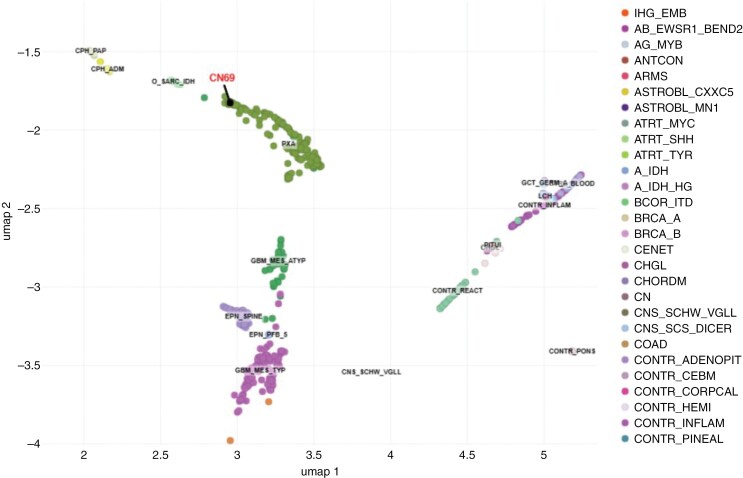
Uniform manifold approximation and projection (UMAP) which revealed a match to the PXA cluster with a high confidence score using both the NCI-EPIC methylation classifier as well as version 12.6 of the Heidelberg classifier.

Due to the difficulty in morphologic classification, the specimen was sent to the National Institutes of Health/National Cancer Institute (NIH/NCI) for DNA methylation profiling, which showed a match to the PXA cluster with a high confidence score of 0.97 using both the NCI-EPIC methylation classifier as well as version 12.6 of the Heidelberg classifier (Figure 4). Although methylation profiling cannot distinguish grades 2 and 3 PXAs, the elevated proliferation index and frequent mitoses supported a grade 3 designation. In addition, next generation sequencing (NGS) by the Mayo Clinic Neuro-Onc Extended Panel was performed and identified a variant of unknown significance in the *ARID2* gene, but did not identify an *NTRK2* rearrangement nor did it test for *BEND5* rearrangement; *BRAF* alterations and *CDKN2A/B* deletions were not detected. A *BEND5-NTRK2* in frame e3:e14 fusion pathogenic Tier 1A variant was detected on the NGS RNA Exome Fusion Panel v2.0 performed at NIH/NCI using custom in-house NGS pipeline (v4.2) to perform fastq file generation (bcl2fastq v2.20.0), alignment and fusion detection; Arriba (v2.2.1) fusion detection tool was used along with STAR (v2.7.10a) aligner for fusion prediction and alignment was done against human genome reference GRCh37/hg19 assembly using Gencode GTF (v36lift37) file for gene annotation of the fusion pairs.The patient was ultimately given the diagnosis of PXA3 with *BEND5-NTRK2* fusion. She began treatment with Larotrectinib. She declined further treatment with radiation and declined additional genetic testing for cancer predisposition syndromes. Serial MRIs have shown no evidence of disease recurrence over the 18 months since Larotrectinib was begun (Figure 2C).

## Discussion

Pediatric patients are at increased risk of glioma development after therapeutic radiation which is age- and dose/volume-dependent.^8^ The Childhood Cancer Survivor Study reported a 8.7 standardized incidence ratio in children previously treated with therapeutic radiation compared to matched controls.^8^ Radiation-induced gliomas (RIG) are typically higher grade, present in atypical sites (eg, suprasellar region, cerebellum), and have a poor prognosis with a median survival of 11 months.^8^ Development of PXA following radiation is rare but has been reported.^9,10^ By definition, RIGs occur in a previously irradiated area, have sufficient latency period, have histology distinct from the primary tumor, and lack a cancer predisposition syndrome.^8^ Our patient’s PXA occurred outside of the targeted field of previous radiation (Figure 1A and B), therefore classifying this tumor as a RIG, particularly in a patient with unknown cancer predisposition status, is speculative at best. However, solid tumors have been reported within a radiation field of 0.5 Gray^11,12^ and secondary CNS malignancies have been reported in volumes that receive <0.1 Gray.^8^ According to our patient’s treatment plan, she received about 1.4 Gy exposure in the likely area of involvement (Figure 1B). Therefore, the effect of this scatter contributing to neoplastic transformation in our patient should be considered.

Survivors of childhood rhabdomyosarcomas have an increased risk of developing a secondary malignant neoplasm (SMN), which has been found to be independent of the use of therapeutic radiation to the primary site.^13^ This suggests that the presence of an underlying predisposition variant is likely contributing to this risk. A retrospective analysis of the surveillance, epidemiology, and end results (SEER) program reported that the majority of SMN were sarcomas, regardless of primary RMS site.^13^ Only two gliomas were reported, however, these were in patients with lower extremity RMS who did not receive any therapeutic radiation.^13^ A large cohort study by the Children’s Oncology Group reported that 7.3% of children with RMS harbored a cancer predisposition variant, of which 10% had embryonal RMS and 3% had alveolar RMS.^14^ As our patient reportedly tested negative for Li-Fraumeni syndrome many years ago, we strongly recommended repeat and expanded testing for cancer predisposition syndromes. Unfortunately, our patient has declined further genetic testing.

Our patient’s brain tumor was difficult to classify based on microscopic features. Diagnoses considered in the histomorphologic differential included the epithelioid variant of PXA3 as well as eGBM, WHO grade 4, an aggressive GBM subtype that carries an average overall survival of 3 months as compared to 31 months in PXA patients.^15–17^The histopathology of PXA3 is often difficult to distinguish from that of eGBM, with whom it shares overlapping cytoarchitectural features, such that PXA3 is sometimes only distinguishable by visualization of focal areas of classical PXA within the tumor tissue.^5^ In addition, they share similar molecular features, including *BRAF V600E* mutations, which are not present in canonical GBMs.^5,16,18^ However, PXA3 typically has better survival than eGBM. Our patient’s final diagnosis of PXA3 is consistent with her favorable outcome thus far.

Fortunately, NGS panel identified an *NTRK2* fusion, which made our patient eligible for targeted treatment with NTRK inhibitor, allowing her to avoid further radiation. The PXA3 in this case harbored a *BEND5-NTRK2* fusion and lacked classical *BRAF* alterations and *CDKN2A/B* deletions typically present in de novo cases. A Neuro-Onc Panel did not identify her *NTRK2* fusion or include testing for *BEND5* alterations. However, an expanded RNA Exome Fusion Panel NGS identified a unique fusion, which made her eligible for targeted treatment. Pediatric oncology providers evaluating rare tumors should be cognizant of the need to do RNA analysis when looking for *NTRK* mutations, as RNA-based NGS methods are superior for detecting *NTRK* fusions as compared to DNA or protein-based assays.^19^

Secondary high-grade gliomas, whether radiation-induced or not, are typically very aggressive with low likelihood of responding successfully to therapy. Despite superior outcomes in treating such cases with further radiation,^8^ our patient declined additional radiation therapy, thereby limiting her treatment options. Our patient is now 18 months from initiation of Larotrectinib without disease progression. This case highlights the importance of expanded NGS testing, particularly in rare tumors. As we continue to expand methylation profiling and molecular testing, we will continue to gain more understanding about these rare tumors allowing for the development of further targeted agents.
